# Harnessing Natural Mosaics: Antibody-Instructed, Multi-Envelope HIV-1 Vaccine Design

**DOI:** 10.3390/v13050884

**Published:** 2021-05-11

**Authors:** Robert E. Sealy, Barry Dayton, David Finkelstein, Julia L. Hurwitz

**Affiliations:** 1 Department of Infectious Diseases, St. Jude Children’s Research Hospital, 262 Danny Thomas Place, Memphis, TN 38105, USA; bob.sealy@stjude.org; 2 Department of Mathematics, Northeastern Illinois University, 5500 N. St Louis Ave, Chicago, IL 60625, USA; bhdayton@neiu.edu; 3 Hartwell Center for Bioinformatics and Biotechnology, St. Jude Children’s Research Hospital, 262 Danny Thomas Place, Memphis, TN 38105, USA; David.finkelstein@stjude.org; 4 Department of Microbiology, Immunology and Biochemistry, University of Tennessee Health Science Center, 858 Madison Avenue, Memphis, TN 38163, USA

**Keywords:** vaccines, protective immunity

## Abstract

The year 2021 marks the 40th anniversary since physicians recognized symptoms of the acquired immunodeficiency syndrome (AIDS), a disease that has since caused more than 30 million deaths worldwide. Despite the passing of four decades, there remains no licensed vaccine for the human immunodeficiency virus type 1 (HIV-1), the etiologic agent of AIDS. Despite the development of outstanding anti-retroviral drugs, there are currently more than one-half million deaths each year due to AIDS. Here, we revisit a conventional vaccine strategy used for protection against variable pathogens like HIV-1, which combines an array of diverse surface antigens. The strategy uses antibody recognition patterns to categorize viruses and their surface antigens into groups. Then a leader is assigned for each group and group leaders are formulated into vaccine cocktails. The group leaders are ‘natural mosaics’, because they share one or more epitope(s) with each of the other group members. We encourage the application of this conventional approach to HIV-1 vaccine design. We suggest that the partnering of an antibody-instructed envelope cocktail with new vaccine vectors will yield a successful vaccine in the HIV-1 field.

## 1. HIV-1 and the Lethal Disease of AIDS

Human immunodeficiency virus type 1 (HIV-1) is the etiologic agent for acquired immunodeficiency syndrome (AIDS), the cause of a public health crisis that has killed approximately 33 million people worldwide. In the year 2019 alone, approximately 38 million people were infected with HIV-1 and approximately 690,000 people died. Effective anti-retroviral drugs have been developed and deployed, but these are not accessed by, or tolerated by, a large fraction of patients. In fact, AIDS-related deaths have been reduced by only 60% since peak deaths in 2004 [1,2]. Despite the passing of several decades since the discovery of HIV-1, there is no licensed vaccine. 

## 2. HIV-1 Vaccine Development—Advanced Clinical Studies

The development of HIV-1 vaccines has followed a long and difficult course. The first vaccine to reach a phase III clinical study was developed by VaxGen Inc. Researchers studied two HIV-1 envelope gp120 proteins, selected to represent viral clades/subtypes of HIV-1 (based on virus sequences) that were common in the geographical regions where clinical studies were performed. No significant protection was observed in clinical studies with one or two gp120 envelope proteins [3,4,5]. 

In response to the failures with envelope-based vaccines, Merck later developed a vaccine that lacked an HIV-1 envelope. This vaccine included only internal proteins and was not designed to induce protective antibodies. In Merck’s phase III study, there was no vaccine-induced protection [6,7].

The RV144 phase III study provided a hint of success [8]. This vaccine included a canary pox-based vector, ALVAC (expressing *env*, *gag*, and *pol* genes), plus two envelope proteins in alum. Using an intent-to-treat analysis of data, study leaders reported that protection was observed at a level of approximately 30% [8]. 

The more recent HIV-1 vaccine phase III studies continue to focus on viral amino acid sequences and clades/subtypes for vaccine design. The Uhambo IIB/III (HVTN 702) study began in 2016 in South Africa and was sponsored by the National Institutes of Health (NIH), USA. One component of the vaccine was an ALVAC canarypox construct. Two additional components were clade/subtype C gp120 envelope proteins formulated with an MF59 adjuvant. Clade/subtype C was targeted, because of its high prevalence in South Africa. When data were evaluated, it was found that 129 HIV-1 infections occurred in the vaccinated group and 123 HIV-1 infections occurred in the placebo control group. Because the vaccine was found to be ineffective, after more than 5400 HIV-1-negative volunteers had been enrolled, NIH and its partners discontinued vaccinations [3,9,10].

There are two large clinical studies ongoing, Imbokodo and Mosaico, supported by the Janssen Pharmaceutical Companies of Johnson & Johnson and the NIH National Institute of Allergy and Infectious Diseases (NIAID). Each study involves the testing of computer-generated, sequence-based mosaics. Imbokodo (HPX2008/HVTN 705) is a Phase IIB study involving a prime-boost with a recombinant adenovirus (Ad26.Mos4.HIV, made by in silico combinations of *env*, *gag*, and *pol* sequences) and a clade/subtype C gp140 envelope protein in adjuvant [3]. The study started in 2017 in southern African countries, finished enrollment of 2600 women in 2019, and is expected to close in 2022 [11,12]. Mosaico (HPX3002/HVTN 706) is a Phase III study designed to enroll approximately 3800 people across eight countries. The Mosaico vaccine components match those of the Imbokodo study, but additionally include a synthetic, sequence-based mosaic gp140 envelope protein. The study began in 2019 and may be completed in 2024 [13,14,15].

To date, the vaccines that have advanced to phase III clinical studies have each included less than a handful of different envelope proteins and have been designed with a focus on the viral amino acid sequences that are common in the geographical locations of the clinical studies. Hundreds of other vaccines have entered pre-clinical or phase I/II clinical studies [3,16,17,18,19,20]. Research has targeted a plethora of delivery vehicles and proteins, protein fragments, or peptides. Proteins have been generated from unmanipulated sequences, mutant sequences, computer generated sequence-based mosaics, consensus sequences, and ancestral sequences. Even a scrambled protein sequence, unlike any natural HIV-1 protein, was the focus of one HIV-1 vaccine program [21].

## 3. Vaccine Successes against Other Diverse Pathogens

HIV-1 is well known for the diversity of its envelope protein, the membrane protein responsible for binding virus to CD4 and other co-receptor molecules on the mammalian cell target. Lessons on how to design successful vaccines against diverse pathogens can be learned from successes in other fields. Routinely, when researchers target diverse pathogens, they use antibody recognition patterns to identify structurally distinct antigens on the pathogen’s surface. For example, when Salk and his colleagues developed the polio vaccine in the mid-1900s, they used antibody reactivity patterns to identify viruses with similar or dissimilar epitopes. They then clustered viruses into three groups based on antibody reactivity patterns (each group included antigenically-similar viruses) and selected a representative from each group for formulation into a vaccine cocktail [22]. The rotavirus vaccine was similarly formulated and includes four components. The Prevnar-13 conjugate vaccine was similarly formulated and includes 13 components; newer pneumococcus vaccines are moving forward from Merck and Pfizer, with 15-valent and 20-valent cocktails, respectively [23]. The papillomavirus vaccine was similarly formulated and has nine components [24]. Each vaccine represents antigenically distinct pathogen variants and is used worldwide with an extraordinary positive influence on human health. 

The immune system comprises B cells, T cells, and innate cells, with each contributing to protective responses against pathogens. B cells and antibodies are often a focus of vaccine development, because when B cells are appropriately triggered, their antibodies can persist lifelong in blood, lymph, and mucosal secretions [25]. When the pathogen initially contacts a host at the point of entry, antibodies serve as a first line of immune defense. 

## 4. Antibody-Instructed HIV-1 Vaccine Design

Here, we consider the use of antibody-instructed cocktail vaccines for the prevention of HIV-1. The strategy is attractive, because: (i) antibodies are prominent defensive weapons against HIV-1 and (ii) many epitopes on viral proteins depend upon three- and four-dimensional (3D and 4D) structures. Epitopes bound by antibodies are hard to predict based on linear sequence alone (although predictions have been improved by recent advances in artificial intelligence [26,27]). When a single amino acid change occurs in a virus, it may be distant from a perceived epitope and may, at first glance, appear irrelevant to antibody binding. In fact, that one change may be sufficient to alter 3D and/or 4D protein structure and abolish antibody binding to a virus [28,29]. Antibody assays, unlike in silico predictions, provide direct evidence that an antibody recognizes its target. 

The concept of designing antibody-instructed envelope cocktails is not new, but the value of these vaccines in the HIV-1 field has not yet been realized. Tests of antibody-instructed HIV-1 envelope cocktails have generally been confined to settings of basic research or phase I clinical trials [17,18,19,20,30,31,32,33,34,35,36]. In past and present years, when HIV-1 research leaders have selected vaccines for advanced clinical trials, they have focused on envelope sequences and clades/subtypes more so than envelope antigenicity [11,12,13,14,15,37].

## 5. Grouping HIV-1 Envelopes by Antigenicity—An Example of Virus/Envelope Selection 

An example of virus/envelope clustering based on antibody reactivity patterns is shown in Table 1. Clustering, in this case, was based on a small dataset described by Moore et al. [38]. Moore et al. had previously performed neutralization assays and reported the reciprocal ID_50_ values (serum dilutions required to neutralize virus at the 50% level) for 21 HIV-1 patient isolates with sera from the same patients. By focusing on a single assay, we avoided errors that could result by mixing data from dissimilar assays. In Table 1A are the original reciprocal ID_50_ values. Viruses and their clades/subtypes are named in columns 1 and 2, respectively, while serum samples are listed on the top row. A score of 0 was given when no neutralization was detected, and a score of 256 was given when the reciprocal ID_50_ was greater than 128. We used a strategy adapted from that of Smith et al. [39], which started with the ranking of data. When the score was 0, the rank was 1; when the score was 8 or 16, the rank was 2; when the score was 32, the rank was 3; when the score was 64 or 128, the rank was 4; when the score was 256, the rank was 5 (Table 1B). The virus with the highest sum of ranked values (92ug001 virus) was assigned as a first leader (L1) for a first virus group (G1). For every other virus, the ranked value for each reciprocal ID_50_ score was subtracted from that of L1 to define distance (d), and the summed distance (sd) was determined. A maximum distance value (Dmax) was chosen (in this case Dmax = 20), and viruses with sd ≤ Dmax were assigned to G1 (indicating similarity of antibody reactivity patterns). Among remaining ungrouped viruses, the virus with the highest sd compared to L1 was selected as a second leader (L2, 92ug024), this time for group 2 (G2). New d and sd values were calculated as before for remaining viruses, this time based on L2 to assign viruses to G2. The process continued iteratively until all viruses were assigned to a group. Using the data in Table 1A and Dmax = 20, the algorithm yielded seven groups (G1–G7) with respective leaders L1–L7 (Table 1B, leaders were 92ug001, 92ug024, 92th022, 92ug037, 92th024, 92th014, and 92rw008). Methods such as this can help researchers recognize viruses/envelopes with similar and disparate patterns of antibody recognition.

The leaders described here are ‘natural mosaics’ because they each contain a composite of epitopes that represent the other envelopes in their group. By using clustering algorithms such as this, and by selecting leaders for assembly, one may begin to create cocktails that represent the antigenic diversity of HIV-1.

How many envelopes can be formulated in a vaccine and recognized by the immune system? The immune system has, in fact, evolved a sophisticated mechanism of antibody production, whereby each developing B cell undergoes a unique gene rearrangement event. The number of unique antibodies that can be made in a human host, and the potential number of unique antigens that can be recognized, may easily exceed one trillion [41]. DNA library vaccines have been described in which thousands of antigens were introduced to the immune system simultaneously and elicited a protective immune response [42]. 

While a small (21 × 21) data matrix was used here, a matrix of any size could be examined for virus/envelope clustering. Mathematica computer code is provided in Supplementary Materials to support analyses of large datasets. There is a vast amount of antibody data in research literature, including data from high-throughput assays, that may assist the visualization of viral antigenic diversity and the design of comprehensive, antibody-instructed, HIV-1 envelope cocktails. 

## 6. Viruses and Antibody Reactivity Patterns Need Not Correlate with Geographical Location

As previously described by Moore et al. [38], and pertinent to vaccine design, the geographical locations of viruses do not define their antibody reactivity patterns [43]. As shown in Table 1, viruses from Thailand often exhibited different antibody reactivity patterns amongst themselves, whereas viruses from different countries (Uganda, Rwanda, Brazil) could sometimes share patterns (Table 1B). 

As an example, two viruses from Thailand, Th024 and TH014, were antigenically distinct. When serum samples were examined for neutralization of these two viruses, scores often differed by ≥2 points in rank; four serum samples recognized Th024 better than Th014, and nine serum samples recognized Th014 better than Th024. If a vaccine were designed to include only one of these two viruses/envelopes, that vaccine would possibly fail to confer protection against the other. If, on the other hand, both viruses were represented in a vaccine cocktail, immune responses to each virus could be generated. 

As another example, most serum samples showed discordant neutralization patterns for the two clade/subtype D viruses, UG005 and UG021. In this case, twelve serum samples recognized UG005 better than UG021, and one serum sample recognized UG021 better than UG005, each with a ≥2-point difference in rank. Again, if a vaccine were designed to include only one of these two viruses/envelopes, the vaccine might fail to confer protection against the other.

Why might viruses within a country differ in antibody recognition patterns, while viruses from different countries share patterns? Immunodeficiency viruses are well known for their high mutation rates. When an individual is first infected with HIV-1, there may be limited viral diversity, but once an immune response is generated toward that virus, a circular evolutionary process begins. Antibodies may clear the founder virus from blood, but virus is maintained in privileged sites where it can generate escape mutants. New viruses induce new B cells and T cells, after which new escape mutants will emerge. Eventually, a plethora of viruses and virus-specific antibodies are present [44,45,46,47]. Because viruses are selected for envelopes that bind conserved mammalian CD4 and co-receptor molecules, shared envelope structures will appear repeatedly across hosts, countries, and clades/subtypes. In essence, a virus is constrained more by its functional requirements than by its geographical location [48]. It is for this reason that the co-evolution of viruses and antibodies in an untreated, infected individual can confer protective immunity against a viral infection from an outside source [7,8,47,49,50,51,52,53,54,55,56,57,58,59]. While the immune system might protect an individual from exogenous virus, it cannot clear endogenous virus from privileged sites (by definition). If the patient is untreated, the chronic virus infection destroys immune cells and eventually destroys the host. A critical aspect of successful vaccination is that the vaccine should be administered before, not after, a virus exposure, so that the immune system can eliminate the virus at its point of entry, prevent virus access to privileged sites, and thereby prevent chronic infection.

## 7. Vaccine Development and Unanswered Questions

### 7.1. Which Assay Should Be Used to Cluster Viruses?

Neutralization data were analyzed in Table 1, but was this the best choice? A clear correlate of protection against HIV-1 has not been defined, meaning that the ‘best’ assay for virus clustering remains a topic of debate. The neutralization assay is often favored, because antibodies are known to protect against immunodeficiency viruses [60,61,62,63] and because the assay measures functional effectors. Nonetheless, neutralization data must be viewed with caution, because protection in vivo does not always predict neutralization in vitro [64]. As an example, the weak protection afforded by the RV144 vaccine did not correlate with neutralization [8,65]. Some assay artifacts might be responsible, at least in part, for failed correlations. Various serum components can mediate non-specific virus kill in the neutralization assay [66]. There are also mutations and unexpected growth characteristics that may arise when viruses are propagated for the preparation of stocks or when chimeric pseudoviruses are used [67,68].

A second choice for use as a virus clustering assay is the enzyme-linked immunosorbent assay (ELISA). There are numerous additional choices to consider, given that virus transmits both by cell-free and cell-to-cell transmission, and that there is an extraordinary number of mechanisms by which antibodies inhibit viruses (e.g., antibody-dependent cellular cytotoxicity [ADCC], antibody-dependent cell-mediated virus inhibition [ADCVI]) [69,70,71,72,73,74]. 

Regardless of the assay chosen, standardization is essential. Attempts have been made to harmonize assays between laboratories [75], but this has not been accomplished globally. Harmonization is perhaps most critical when data are combined into banks (e.g., CATNAP [76]). Strict attention to data origin may help avoid misinterpretations and inaccurate virus comparisons. 

### 7.2. Which Antibody Source Should Be Used? 

In many vaccine fields, serum antibodies are used to identify virus groups. Human serum antibodies work well when a pathogen infection is acute, because each human sample will exhibit limited responsiveness. In HIV-1-infected patients, however, because of the chronic nature of infection, there is ongoing virus-antibody evolution as described above [46]. When serum antibodies are taken at a late stage of infection, they may recognize too many viruses to be useful for virus discrimination [36]. Perhaps a combination of monoclonal antibodies, patient sera, and polyclonal antibodies generated in research animals after controlled envelope immunizations will best serve as tools for antibody-instructed HIV-1 envelope clustering [77].

### 7.3. What Should Be the Dmax?

The selection of Dmax must weigh many factors. As Dmax increases, the representation of antigenic diversity in a vaccine is diminished, a situation that can lead to pathogen escape from a vaccine-induced immune response [28,78,79]. As Dmax decreases, cocktails will increase in size, as will vaccine manufacturing and regulatory requirements. Presently, pneumococcus and papillomavirus vaccines are increasing in complexity, each with positive results [23,24,80]. New vaccine delivery vehicles are being developed that may simplify manufacturing logistics and support the development of complex cocktail vaccines, both within and outside of the HIV-1 field. 

### 7.4. Which Vaccine Delivery Vehicle Should Be Used?

Once a cocktail is selected, what vaccine vehicle should be used? There have been dozens of delivery vehicles studied in the HIV-1 field, most of which can support the envelope cocktail approach. During the past year, mRNA vaccines have emerged as attractive delivery vehicles for severe acute respiratory syndrome coronavirus-2 (SARS-CoV-2) vaccines [81,82]. mRNA vaccines might now facilitate HIV-1 vaccine development. The use of mRNA may simplify the logistics of cocktail vaccine development and thereby support an increase in cocktail complexity. mRNA vaccines are additionally attractive in that they, like live viral vaccines [17,83,84,85], instruct the endogenous expression of antigens. Endogenous antigen expression, in turn, supports the robust activation of cytotoxic T lymphocytes (CTLs) that provide a fail-safe mechanism by clearing virus-infected cells if/when antibodies are not fully protective. The situation differs from that of subunit vaccines that require antigen cross-presentation to trigger CTLs [86,87] and that generally elicit relatively weak CTL responses. mRNA and live viral vaccines associate with durable immune responses that can persist for months, years, or decades [17,25,88,89]. Each of these qualities may increase the efficacy of an HIV-1 vaccine.

### 7.5. Which Gatekeepers for Clinical Vaccine Development Should Be Used? 

Once a new HIV-1 vaccine candidate is designed, animal research studies can provide critical information about immunogenicity. The vaccination of small animals is a routine method for measuring the magnitude and quality of vaccine-induced B cell and T cell immune responses. A next step in HIV-1 vaccine development often involves the vaccination and challenge of non-human primates. Decades ago, chimpanzees were chosen for non-human primate experiments, because chimps were susceptible to HIV-1 infections. Today, challenges are usually performed in macaques. Instead of using HIV-1, scientists create chimeric viruses by combining sequences from HIV-1 and simian immunodeficiency viruses (SHIVs). Chimeras are then passaged repeatedly in monkeys to select for pathogenic strains [90]. SHIVs are not a perfect model for HIV-1 [47], and vaccines that successfully protect against SHIV challenges have failed in human clinical trials. By the same token, a vaccine that protects humans from HIV-1 might fail in a SHIV study. The question therefore remains open as to which gatekeeper is best for advancing HIV-1 vaccines to clinical trials.

### 7.6. Vaccine Development in the Context of Unanswered Questions

Many questions remain in the HIV-1 field that can easily stymy vaccine development. The situation is not unique, as every vaccine field must grapple with imperfect systems and unanswered questions. In the mid-1900s, Macfarlane Burnet, who later won a Nobel Prize for his discovery of clonal selection, contemplated that a successful polio vaccine might never be developed [91]. Soon thereafter, Salk and colleagues forged ahead despite unanswered questions, and with support from community leaders and the March of Dimes, developed a successful vaccine [92,93]. In the pneumococcus field, antibody-instructed vaccines are constantly being questioned and improved, yet licensed vaccines continue to save lives. In the coronavirus disease 2019 (COVID-19) field, with intense public support despite unanswered questions, scientists demonstrated unprecedented speed in the development of SARS-CoV-2 vaccines [81,82,89,94]. Perhaps the time is opportune to accelerate activities in the HIV-1 research field, partner natural mosaics with novel vectors, and develop a vaccine that can prevent AIDS. 

## 8. Conclusions

Our emphasis here is that multi-envelope vaccine design can be instructed by antibody reactivity patterns. Antibody-instructed vaccine cocktails have already succeeded in numerous fields. In the HIV-1 field, the partnering of new vectors (e.g., mRNA), data from antibody assays, and clustering algorithms might now expedite the design of a cocktail vaccine. A successful vaccine product may then be advanced to combat the ongoing public health crisis of HIV-1 and AIDS.

## Figures and Tables

**Table 1:** Clustering viruses by antibody recognition patterns. (**A**) Data are from a neutralization experiment by Moore et al. [38], from which ID_50_ scores were recorded. Viruses and clades/subtypes are listed in the first two columns, and sera are listed as headers of additional columns. The year and origin of virus isolation is defined in the name (TH = Thailand, UG = Uganda, RW = Rwanda, BR = Brazil). Clade/subtype “E” is now recognized as a circulating recombinant form AE [40]. Each entry is ID_50_, with ‘0‘ representing no neutralization, and ‘256’ representing an ID_50_ greater than 128. (**B**) Values from Table 1A were ranked from 1 to 5, and ranks were color-coded using Microsoft Excel software (Microsoft Corporation, Remond, Washington, USA). Viruses were grouped based on similar antibody reactivity patterns using Dmax = 20 (viruses with summed differences ≤ Dmax compared to a leader were assigned to the same group). There were seven groups (separated by thick black lines) with respective leaders indicated at the top of each group in bold type.

**A. Original Data**																						
**VIRUSES**	**VIRUS CLADE**	**ID50 WITH HUMAN SERUM SAMPLES:**																				
Virus		92UG029	92UG031	92UG037	92RW009	92RW008	92TH014	92TH 026	92BR 020	92BR 021	92BR 023	92BR025	92UG001	92UG005	92UG021	92UG046	92UG024	92TH006	92TH009	92TH022	92TH024	92TH023
_92ug029	A	0	256	0	0	32	256	256	0	0	64	256	256	256	64	32	256	128	0	256	32	256
_92ug031	A	16	16	128	8	64	8	16	0	0	32	8	16	128	16	0	0	8	32	64	8	0
_92ug037	A	0	32	128	8	256	0	0	0	0	128	64	0	32	0	0	0	0	64	64	0	0
_92rw009	A	0	32	64	8	256	16	0	0	0	32	64	0	64	0	0	0	0	32	32	8	0
_92rw008	A	0	128	128	128	32	0	8	0	0	128	32	128	128	128	128	0	0	0	8	0	0
_92th014	B	128	16	128	8	128	128	128	128	128	128	128	0	128	0	0	8	0	8	128	64	8
_92th026	B	0	0	0	0	32	8	0	0	0	0	0	8	256	0	0	0	0	0	8	0	0
_92br020	B	32	0	0	0	32	0	16	0	0	0	0	0	128	0	0	0	0	0	0	16	0
_92br021	B	0	0	0	0	32	0	8	16	8	8	0	0	128	0	0	0	0	8	0	8	0
_92br023	B	0	64	32	64	128	0	0	0	128	0	128	16	128	0	0	0	0	16	128	0	0
_92bf025	C	0	16	0	0	128	0	0	0	0	8	8	0	0	0	0	0	0	0	8	0	0
_92ug001	D	8	256	8	0	64	64	256	8	0	64	256	256	256	32	8	256	128	8	256	256	256
_92ug005	D	0	64	64	64	256	64	64	0	0	256	64	32	256	0	8	0	0	128	256	16	0
_92ug021	D	0	0	0	0	8	0	0	0	0	0	0	0	8	0	0	0	0	0	0	256	0
_92ug046	D	0	0	0	0	8	0	0	0	0	0	0	0	128	8	0	0	0	0	0	0	0
_92ug024	D	0	0	0	0	32	0	0	0	0	0	0	0	16	0	0	0	0	0	0	0	0
_92th006	“E”	0	8	128	128	32	128	16	64	0	0	128	0	32	128	128	64	128	32	0	0	128
_92th009	“E”	0	16	32	128	16	128	32	16	0	0	128	0	8	128	64	8	64	16	16	0	128
_92th022	“E”	0	128	128	128	32	128	64	128	0	0	128	8	128	128	128	64	128	32	0	0	128
_92th024	“E”	0	16	32	128	16	128	0	128	0	0	16	0	16	128	32	0	8	0	16	0	128
_92th023	“E”	0	8	64	128	32	128	0	16	0	0	16	0	8	128	128	8	16	8	0	0	128
**B. Grouped viruses**																						
**VIRUSES**		**RANKED SCORES WITH HUMAN SERUM SAMPLES:**																				
		92UG029	92UG031	92UG037	92RW009	92RW008	92TH014	92TH026	92BR020	92BR021	92BR023	92BR025	92UG001	92UG005	92UG021	92UG046	92UG024	92TH006	92TH009	92TH022	92TH024	92TH023
**_92ug001**	**D**	2	5	2	1	4	4	5	2	1	4	5	5	5	3	2	5	4	2	5	5	5
_92ug029	A	1	5	1	1	3	5	5	1	1	4	5	5	5	4	3	5	4	1	5	3	5
																						
**_92ug024**	**D**	1	1	1	1	3	1	1	1	1	1	1	1	2	1	1	1	1	1	1	1	1
_92th026	B	1	1	1	1	3	2	1	1	1	1	1	2	5	1	1	1	1	1	2	1	1
_92br020	B	3	1	1	1	3	1	2	1	1	1	1	1	4	1	1	1	1	1	1	2	1
_92br021	B	1	1	1	1	3	1	2	2	2	2	1	1	4	1	1	1	1	2	1	2	1
_92br025	C	1	2	1	1	4	1	1	1	1	2	2	1	1	1	1	1	1	1	2	1	1
_92ug021	D	1	1	1	1	2	1	1	1	1	1	1	1	2	1	1	1	1	1	1	5	1
_92ug046	D	1	1	1	1	2	1	1	1	1	1	1	1	4	2	1	1	1	1	1	1	1
																						
**_92th022**	“E”	1	4	4	4	3	4	4	4	1	1	4	2	4	4	4	4	4	3	1	1	4
_92th006	“E”	1	2	4	4	3	4	2	4	1	1	4	1	3	4	4	4	4	3	1	1	4
_92th009	“E”	1	2	3	4	2	4	3	2	1	1	4	1	2	4	4	2	4	2	2	1	4
_92th023	“E”	1	2	4	4	3	4	1	2	1	1	2	1	2	4	4	2	2	2	1	1	4
																						
**_92ug037**	**A**	1	3	4	2	5	1	1	1	1	4	4	1	3	1	1	1	1	4	4	1	1
_92ug031	A	2	2	4	2	4	2	2	1	1	3	2	2	4	2	1	1	2	3	4	2	1
_92rw009	A	1	3	4	2	5	2	1	1	1	3	4	1	4	1	1	1	1	3	3	2	1
_92ug005	D	1	4	4	4	5	4	4	1	1	5	4	3	5	1	2	1	1	4	5	2	1
_92br023	B	1	4	3	4	4	1	1	1	4	1	4	2	4	1	1	1	1	2	4	1	1
																						
**_92th024**	“E”	1	2	3	4	2	4	1	4	1	1	2	1	2	4	3	1	2	1	2	1	4
																						
**_92th014**	**B**	4	2	4	2	4	4	4	4	4	4	4	1	4	1	1	2	1	2	4	4	2
																						
**_92rw008**	**A**	1	4	4	4	3	1	2	1	1	4	3	4	4	4	4	1	1	1	2	1	1

## Data Availability

Not applicable.

## Supplementary Materials

The following are available online at https://www.mdpi.com/article/10.3390/v13050884/s1
