# Supplementary material for: Harnessing Natural Mosaics: Antibody-Instructed, Multi-Envelope HIV-1 Vaccine Design
Source: Viruses. 2021 May 11;13(5):884. doi: 10.3390/v13050884 (PMC8151930; doi:10.3390/v13050884)
Supplement: Supplementary file 1 [file viruses-13-00884-s001.zip › viruses-1147507-supplementary.pdf]

Supplementary Method

Software for grouping viruses and selecting leaders: We have developed software to cluster viruses and assign leaders as shown in Table 1. To serve the largest audience, the data input is in the form of a .CSV file. The data in each row, a vector, is separated by commas. Rows are marked by line break characters. The first entry is an alphanumeric identifier (e.g. 92ug001 virus). The remaining entries must be numeric, whole numbers or decimals. The software will accommodate large .csv files on desktop or cloud.

The user must designate a distance function, on the vectors. Generally, for this purpose we will sum the absolute values of the difference in coordinates; this distance function is designated by the number 1. Euclidean distance, the square root of the sum of differences squared, is designated by the number 2. Finally, the parameter Dmax must be given.

The software is written in the *Mathematica* language, (trademark and copyright owned by Wolfram Research, Inc.), appropriate for combining literal and mathematical information. The output is in a form that one can cut and paste into a form as in Table 1b. Code for a laptop computer is given here:

```
vaGroups4CSV[F_,norm_,maxd_]:=Module[{dim,F1,A,l,R,used,comp,leaders,sel,c,mi
nDistA,distA,groups},
(* Read in CSV File and check  *)
      dim=Dimensions[F];
      If[Length[dim]!=2,Print["Dimension error in CSV"];Abort[]];
      F1=Take[F,All,-dim[[2]]+1];
      If[!MatrixQ[F1,NumberQ],Print["Non-numeric entry"];Abort[]];
      If[!NumberQ[norm]||norm<1,Print["Invalid norm"];Abort[]];
(* Form Association, define distances *)
      A=Association[Table[F[[i,1]]->F1[[i]],{i,dim[[1]]}]];
      R=Keys[A];
      l=Length[R];
      distA[x_,y_]:=Norm[A[x]-A[y],norm];
      minDistA[S_,x_]:=Min[Table[distA[a,x],{a,S}]];
(* Find First Leader *)
      c=SortBy[Table[{x,Norm[A[x],norm]},{x,R}],Last][[-1,1]];
      leaders={c};
      used={c};
      comp=Complement[R,used];
```

```
      sel[c]=Prepend[Select[comp,distA[c,#]<=maxd&],c];
      used=sel[c];
      comp=Complement[R,used];
      groups={sel[c]};
(* Main loop *)
      While[Length[used]<l,
      c=SortBy[Table[{x, minDistA[{leaders[[-1]]},x]},{x,comp}],Last][[-1,1]];
      AppendTo[leaders,c];
      AppendTo[used,c];
      comp=Complement[R,used];
      sel[c]=Prepend[Select[comp,distA[c,#]<=maxd&],c];
      used=Union[used,sel[c]];
      comp=Complement[R,used];
      groups=Append[groups,sel[c]]];
(* Output answer *)
      Column[{{"Number of Groups",Length[leaders]},{},
      Column[Table[Column[Prepend[Table[Flatten[{x,A[x]}],
      {x,groups[[i]]}],leaders[[i]]]],{i,Length[groups]}]]}]]
```
